# Cyclin B Translation Depends on mTOR Activity after Fertilization in Sea Urchin Embryos

**DOI:** 10.1371/journal.pone.0150318

**Published:** 2016-03-10

**Authors:** Héloïse Chassé, Odile Mulner-Lorillon, Sandrine Boulben, Virginie Glippa, Julia Morales, Patrick Cormier

**Affiliations:** 1 Sorbonne Universités, UPMC Univ Paris 06, UMR 8227, Integrative Biology of Marine Models, Translation Cell Cycle and Development, Station Biologique de Roscoff, CS 90074, F-29688, Roscoff cedex, France; 2 CNRS, UMR 8227, Integrative Biology of Marine Models, Station Biologique de Roscoff, CS 90074, F-29688, Roscoff cedex, France; University of Siena, ITALY

## Abstract

The cyclin B/CDK1 complex is a key regulator of mitotic entry. Using PP242, a specific ATP-competitive inhibitor of mTOR kinase, we provide evidence that the mTOR signalling pathway controls *cyclin B* mRNA translation following fertilization in *Sphaerechinus granularis* and *Paracentrotus lividus*. We show that PP242 inhibits the degradation of the cap-dependent translation repressor 4E-BP (eukaryotic initiation factor 4E-Binding Protein). PP242 inhibits global protein synthesis, delays cyclin B accumulation, cyclin B/CDK1 complex activation and consequently entry into the mitotic phase of the cell cycle triggered by fertilization. PP242 inhibits *cyclin B* mRNA recruitment into active polysomes triggered by fertilization. An amount of *cyclin B* mRNA present in active polysomes appears to be insensitive to PP242 treatment. Taken together, our results suggest that, following sea urchin egg fertilization, *cyclin B* mRNA translation is controlled by two independent mechanisms: a PP242-sensitive and an additional PP242-insentitive mechanism.

## Introduction

Cyclins and their catalytic kinase partners CDKs (Cyclin-Dependent Kinases) control cell cycle progression [1]. The mitotic cyclins A and B were first discovered in sea urchin as key proteins, which are synthetized and degraded during M-phase at each cell division [2]. Different mechanisms that underlie the control of mitotic cyclin B translation during the cell cycle and development transitions of model organisms have been reported [3]. In sea urchin and clams, cyclin B is synthetized continuously and rapidly destroyed shortly before the metaphase-anaphase transition of the mitotic cell cycles [2]. In *Xenopus*, cyclin B translation was reported to be maximal during mitosis and to be driven by the polyadenylation of *cyclin B* mRNA in cycling extracts from embryos [4]. CDK1-mediated negative feedback loop was shown to decrease cyclin B translation and drive *Xenopus* early embryonic cell cycle oscillations [5]. Taken together, these studies highlight the requirement for fine-tuning of mitotic cyclins translation during the cell cycle [3, 6, 7].

The early steps of the first mitotic division induced by fertilization of sea urchin egg represent an optimal system for studying the relationships between mRNA translation regulation and mitotic cell cycle for the following reasons. Sea urchin eggs have completed their meiotic maturation and are haploid cells blocked in G1. The overall rate of protein synthesis is low in unfertilized eggs and fertilization triggers a dramatic rise in protein synthesis independently of mRNA transcription [8]. Sea urchin embryos are naturally synchronized during the first mitotic divisions. *De novo* protein synthesis is dispensable for S-phase progression but is required for the onset and normal progression of mitosis [9]. A comparative genomics platform for the echinoderm clade is available (http://Echinobase.org [10]). The *S*. *purpuratus* genome analysis allowed identifying the canonical B-type cyclin [11, 12]. Cap-dependent translation is highly regulated following fertilization and is involved in the onset of the first mitotic division of the sea urchin embryos [13–15].

The mechanism of eukaryotic cap-dependent initiation of translation accounts for the translation of most eukaryotic mRNAs [16]. Eukaryotic Initiation Factor 4E (eIF4E), a central actor for the control of cap-dependent translation initiation, is a cap-binding protein that binds to m^7^GpppN (where N is any nucleotide) present at the 5’ extremity of the vast majority of eukaryotic mRNAs (review in [17]). eIF4E bridges the mRNA and the ribosome [18] by recruiting eukaryotic Initiation Factor 4G (eIF4G), a scaffolding protein that acts as docking site for several proteins including Initiation Factors 3 (eIF3) and 4A (eIF4A). eIF4G also associates with the Poly(A)-Binding Protein (PABP) that interacts with poly(A) tail. This association stimulates translation of polyadenylated mRNAs [19].

eIF4E-binding proteins (4E-BPs) are well-characterized inhibitors of eIF4E function (review in [20]). Three 4E-BPs (4E-BP1, 4E-BP2 and 4E-BP3) exist in mammals [21–23]. A single 4E-BP ortholog exists in sea urchin [24]. 4E-BPs competitively inhibit eIF4G association with eIF4E. Binding of 4E-BPs to eIF4E is regulated by phosphorylation [25, 26]. Hypophosphorylated 4E-BPs bind to eIF4E and inhibit cap-dependent translation, whereas hyperphosphorylated forms do not.

4E-BPs phosphorylation is controlled *via* the mTOR (mechanistic Target Of Rapamycin) pathway (reviews in [27, 28]). mTOR is an atypical serine/threonine protein kinase that interacts with several proteins to form two distinct complexes named mTOR Complex 1 (TORC1) and 2 (TORC2), which are evolutionarily conserved from yeast to mammals. Both complexes have been implicated in the regulation of cell growth in response to various stimuli, TORC1 controlling the cell mass whereas TORC2 would be responsible for cell surface area control [29]. Translational regulation is the best characterized process regulated by TORC1, which phosphorylates p70 ribosomal S6 protein kinase and 4E-BP1, which in turn, promote protein synthesis (review in [30]).

We previously showed in the sea urchin *Spharechinus granularis* that 4E-BP degradation triggered by fertilization allows eIF4E association with eIF4G and the consequent accumulation of cyclin B [31, 32]. 4E-BP degradation induced by fertilization of sea urchin eggs is affected by rapamycin treatment, suggesting that it reflects TORC1 signalling [31]. Rapamycin does not directly inhibit TOR kinase activity [33]. In mammal cells, rapamycin associates with FKBP12 (12-kDa FK506 binding protein) and together they affect TOR enzymatic activities by binding to a domain different from mTOR kinase site.

PP242 is a novel and specific ATP-competitive inhibitor of mTOR kinase [34]. PP242 is a dual inhibitor of TORC1 and TORC2 and it inhibits more efficiently TORC1 than rapamycin. Here, using PP242, we set out to study mTOR signalling in the control of mitotic cyclin B translation following egg fertilization in *S*. *granularis* and *P*. *lividus*, two sea urchin species separated by 20 million years evolution [10, 35]. We show that PP242 inhibits drastically 4E-BP degradation and delays cyclin B accumulation triggered by fertilization. PP242 consequently delays cyclin B/CDK1 complex activation and mitotic cell division triggered by fertilization. We demonstrate that *cyclin B* mRNA recruitment into active polysomes induced by fertilization is controlled by a PP242-sensitive pathway. Using puromycin as an active polysome disrupter, our results suggest that an additional PP242-insensitive mechanism is involved in the control of *cyclin B* mRNA recruitment into polysomes.

## Materials and Methods

### Chemicals

Sodium orthovanadate, EDTA, EGTA, dithiothreitol (DTT), N -2-hydroxyethylpiperazine-N’-2-ethanesulfonic acid (Hepes), sodium fluoride, p-nitrophenyl phosphate, leupeptin, aprotinin, soybean trypsin inhibitor, benzamidine, ATP, Tween 20, puromycin, emetine, and Triton X-100 were obtained from Sigma-Aldrich (France).

[^35^S]L-methionine, was purchased from Perkin–Elmer (France). Rabbit polyclonal antibodies directed against *S*. *granularis* cyclin B [36]) were a generous gift from Professor Gérard Peaucellier (Banyuls, France). Rabbit polyclonal antibodies directed against *S*. *granularis* 4E-BP were previously described [37]. The mTOR inhibitor PP242 and mouse monoclonal antibody directed against human PSTAIR (P7962) were purchased from Sigma-Aldrich (France). Rabbit monoclonal antibody directed against human Phospho^320^T-PP1Cα (ab62334) [38] was obtained from Abcam. Mouse monoclonal antibody directed against rabbit eIF4E was purchased from Transduction Laboratories (Lexington, KY). Horseradish peroxidase-conjugated secondary antibodies were obtained from Dako SA. Amersham ECL Western blotting detection reagents were from GE Healthcare and ECL2 Western blotting substrate was from Pierce.

### Handling of gametes and embryos

*Sphaerechinus granularis* and *Paracentrotus lividus* collected in the Brest area (France), were obtained from CRBM (Centre de Ressources Biologiques Marines) at the Roscoff Biological Station (France). Spawning of gametes was induced by intracoelomic injection of 0.1 M acetylcholine. Eggs were collected in 0.22 μm Millipore-filtered seawater (FSW) and rinsed twice by centrifugation (2,000 rpm, 2 min). Eggs were dejellied by swirling twenty seconds in 3.5 mM citric acid pH5 and rinsed three times with fresh FSW. For fertilization, eggs were suspended in FSW (5% suspension). Diluted sperm was added to the eggs. Experiments were only performed on batches exhibiting greater than 90% fertilization, each experiment used gametes from a single female. Cultures were performed at 16°C under constant agitation. When required PP242 was added at the indicated final concentration to the egg suspension 10 min before fertilization from a 30 mM stock solution in DMSO. For polysome analyses, in order to freeze ongoing translating polysomes, emetine was added to the embryos suspension 5 min prior lysis. When required, disruption of translating polysomes was performed *in vivo* by adding puromycin (0.6 mM final concentration) to the embryos suspension 20 min prior lysis for polysomal RNA preparation.

### Determination of cleavage rates and cytological analysis

At time intervals during 4 hours after fertilization, cleavage was scored by observation under a phase contrast microscope. Thousands of embryos were incubated for each experimental determination, from which around 100 were scored for cleavage occurrence. At various times after fertilization, 0.2 ml aliquots of embryo suspension were fixed overnight in 1 ml methanol/glycerol (3:1, v/v) in the presence of the DNA dye Hoechst (bisbenzimide, 0.1 μg/ml), mounted in 50% glycerol. The nuclear envelope and DNA were respectively observed under Nomarski differential interference contrast (DIC) and fluorescence microscopy (Zeiss, Marly Le Roi, France).

### Embryo extracts and Western blot analyses

At different times following fertilization, total extracts were obtained by direct solubilization of 20 μl pelleted cells (eggs or embryos) in 150 μl of SDS-Fix buffer containing 2% sodium dodecyl-sulfate (SDS), 10% glycerol, 5% β-mercaptoethanol, 62.5 mM Tris HCl pH 6.8.

Proteins were resolved by SDS-PAGE. 4E-BP level was assessed after sample resolution on a gel containing 15% acrylamide/bisacrylamide from a 40% stock solution (acrylamide/bis acrylamide; 2.6%), and Phospho-PP1Cα (^320^T in human sequence; ^318^T in sea urchin sequences, S1 Fig) and cyclin B analyses were performed on a 12% acrylamide gel containing 0.1% bisacrylamide.

Western blot analyses were performed following electrophoretic transfer of proteins from SDS-PAGE onto 0.22 μm nitrocellulose membranes [39]. Membranes were incubated with antibodies against cyclin B (1:1000), 4E-BP (1:5000), phospho-PP1Cα (1:1000), PSTAIR (1:1000), eIF4E (1:2000). The antigen-antibody complex was measured by chemiluminescence using horseradish peroxydase-coupled secondary antibodies according to the manufacturer’s instructions (ECL or ECL2). Signals were quantified using the public domain ImageJ program (written by Wayne Rasband at the US National Institutes of Health, Bethesda, Maryland, USA, http://imagej.nih.gov/ij/, 1997–2015.).

### Analysis of cyclin B/CDK1 complex activity *in vivo*

The activation state of cyclin B/CDK1 complex was determined by monitoring the endogenous phosphorylation status of PP1Cα (^320^T in human sequence; ^318^T in sea urchin sequences, S1 Fig), the catalytic subunit of protein phosphatase 1 shown to be a natural substrate for CDK1 [38]. Measurements were done by Western blotting after electrophoretic resolution of total protein extracts from embryos taken at indicated times after fertilization.

### Protein synthesis *in vivo*

A batch of unfertilized eggs (5% suspension in FSW) was incubated for 1 h in 10 μCi/ml [^35^S]-L-methionine at 16°C. Eggs were then rinsed in FSW and fertilized in the presence or absence of 10 μM PP242. At the indicated times, 200 μl of cell suspension was pelleted and frozen in liquid nitrogen. Cell extracts were prepared by re-suspending the pellets in 400 μl ice-cold buffer (40 mM HEPES pH7.6, 100 mM NaCl, 0.4 mM EDTA, 2 mM dithiothreitol (DTT), 0.2 mM Sodium orthovanadate, 20 mM *p*-nitrophenyl phosphate, 100 mM sodium fluoride, 100 mM β-glycerophosphate, and 10 μg/ml Protease Inhibitor Cocktail (P2714, Sigma-Aldrich). [^35^S]methionine incorporation was measured on duplicate aliquots after 10% TCA precipitation on Whatman 3M filters and counting in the presence of Optiphase Supermix scintillation liquid. Radioactive proteins were visualized after resolution on 12% acrylamide SDS-PAGE followed by autoradiography of the gels on Kodak Biomax MR films.

### Polysomal RNA preparation, RNA extraction and RT-PCR analysis

5 ml of 5% cell suspension was pelleted. Pelleted cells were lysed with a Dounce homogenizer in 1 ml of polysome lysis buffer in RNase-free water (10 mM Tris pH 7.4; 250 mM KCl; 10 mM MgCl2; 25 mM EGTA; 0.4% Igepal; 5% sucrose; 1 mM DTT; 10 μg/μl Aprotinin; 2 μg/ml Leupeptin; 100 μg/ml Emetine; 40U RNase inhibitor). Lysates were then clarified for 10 min at 13,000 rpm. Supernatants were loaded on a linear 15–40% sucrose gradient (10 mM Tris pH 7.4; 250 mM KCl; 10 mM MgCl2; 25 mM EGTA; 1 mM DTT), which ran for 2.5h at 38,000 rpm in a SW41Ti rotor at 4°C. Gradients were fractionated into equal fractions (~600 μl per fraction). RNAs were extracted with the KingFisher Pure RNA tissue kit (ThermoFisher), each fraction was diluted with one volume lysis buffer provided in the kit and processed using manufacturer’s instruction. RNA integrity was checked on 2% agarose/TBE gel electrophoresis. Equal volume of RNA isolated from each fraction was used for cDNA synthesis using the reverse transcriptase SuperScript II (Invitrogen) according to manufacturer’s instruction. cDNAs were diluted in RNase-free water (1 vol. RT/300 vol. H_2_O). Amplicon amplifications were done using the GoTaq Flexi kit (Promega). Primers were designed from *S*. *granularis* cyclin B sequence (Y08016): F (5’-GCCAGCAAGTATGAAGAG-3’) and R (5’-AACCTCCATCTGTCTGAT-3’) or *P*. *lividus* cyclin B sequence (http://octopus.obs-vlfr.fr/): F (5’-CAAAGAGCATGGCTGTTCAA-3) and R (5’-CCATTGTATCCATCGCCTCT-3’) and were used at 0.6 μM final concentration. PCR were carried out as followed: 95°C for 2 min; followed by 30 cycles of 3 steps: 95°C for 30 s, 60°C for 30 s, 72°C for 1 min; and finally 72°C for 5 min. PCR products were analyzed on 2% agarose/TBE gels electrophoresis and scanned on a Typhoon Trio (GE Healthcare Life Sciences). Signals were quantified using the public domain ImageJ program (written by Wayne Rasband at the US National Institutes of Health, Bethesda, Maryland, USA, http://imagej.nih.gov/ij/, 1997–2015.).

## Results

### PP242 affects early embryonic development in sea urchins

**Fig 1:**
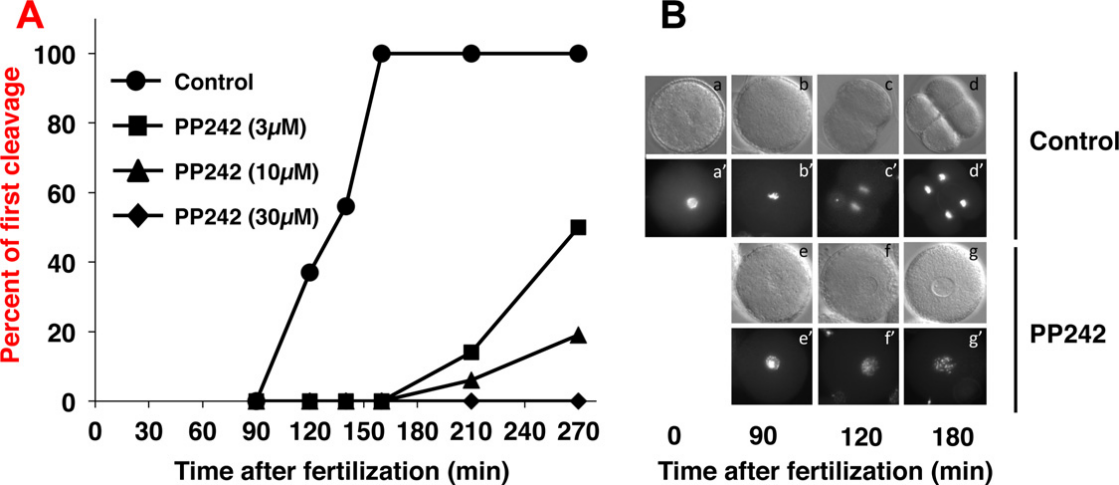
PP242 affects the first mitotic division of *S*. *granularis* embryos. **(A)** Dose-response effect of PP242 on the first mitotic division of sea urchin early development. Batches of *S*. *granularis* eggs (5% cells/vol solution in FSW) were pre-incubated 10 min before sperm addition with DMSO (circles) or 3 μM (squares), 10 μM (triangles) or 30 μM (diamonds) PP242. Cleavage rates were scored at different times during the time culture in the continuous presence of the drug. The curve was obtained from the eggs isolated from a single female and was representative of 8 independent experiments. **(B)** Microscopic observation of nuclear envelope (a-g) and chromatin (a’-g’) morphology in control (a-d and a’-d’) and 10 μM PP242-treated embryos (e-g and e’-g’) at the indicated times post-fertilization.

The effect of PP242 was first analysed on the kinetics of cell division following fertilization. Unfertilized *S*. *granularis* eggs were pre-incubated for 10 min in the presence of different PP242 concentrations. They were then fertilized, cultured in drug-containing medium and scored for the first embryonic division (Fig 1A). PP242 induced a dose-dependent delay in the occurrence of cell division as judged by the percent of first cleavage measured during 4 hours post fertilization. At 30 μM the drug completely prevented the resumption of cell cycle induced by fertilization. At 10 μM PP242, at the time when first cleavage had occurred in the totality of control embryos, none of the treated embryos displayed cell division. Some divisions occurred then after in PP242-treated embryos, never reaching 100% of the batch and with major cell cycle di-synchrony as judged by changes in the slope of the curve. This PP242-induced delay in the first division was reproducibly obtained in 8 different females, ranging from one hour to more than 3 hours. PP242 did not affect fertilization *per se* since elevation of the fertilization membrane and the movement of the female and male pronuclei up to their fusion occurred normally (Fig 1B). However, the nuclear envelope remained intact until at least 180 min post fertilization indicating a block at the entry into M-phase of the cell cycle.

**Fig 2:**
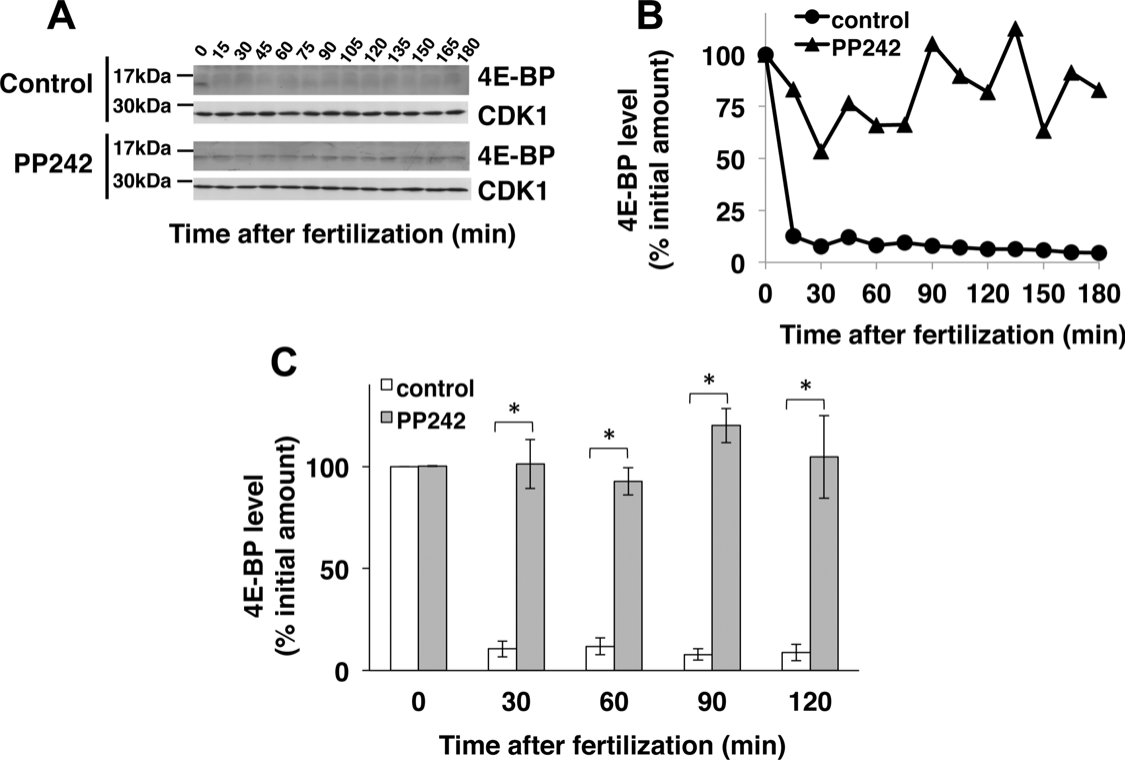
PP242 inhibits 4E-BP degradation triggered by fertilization. **(A)** Total amount of 4E-BP in extracts from control or PP242-treated embryos of *S*. *granularis* was analysed by Western blotting using anti-sea urchin 4E-BP antibodies (top panels). CDK1 immunolabelling with PSTAIR antibody was used as a loading control for Western blot (bottom panels). **(B)** Quantification of the results obtained (in (A)) from control (circles) or PP242 treated (triangles) embryos. 4E-BP amount was normalized against CDK1 level and expressed as a percentage of the value obtained with unfertilized eggs. **(C)** Quantitation of 4E-BP abundance obtained from untreated (white boxes) and PP242-treated (grey boxes) embryos at the indicated time following fertilization. 4E-BP amount was normalized against CDK1 level and expressed as a percentage of the value obtained with unfertilized eggs. Vertical bars represent Standard Error of the Mean values (SEM) obtained in 6 independent experiments. (*) Indicates a significant difference in 4E-BP amount in PP242-treated embryos in comparison to the untreated embryos (Wilcoxon signed-rank test, p<0.05).

The main well-known target of mTOR in cells is the cap-dependent protein synthesis inhibitor, 4E-BP. The effect of PP242 on 4E-BP was therefore examined (Fig 2). As already reported [31] fertilization induced the rapid and almost complete disappearance of 4E-BP during the first 15 minutes. Fertilization in the presence of PP242 completely inhibited 4E-BP disappearance, the protein remaining at a constant and high level for more than 180 min after fertilization.

**Fig 3:**
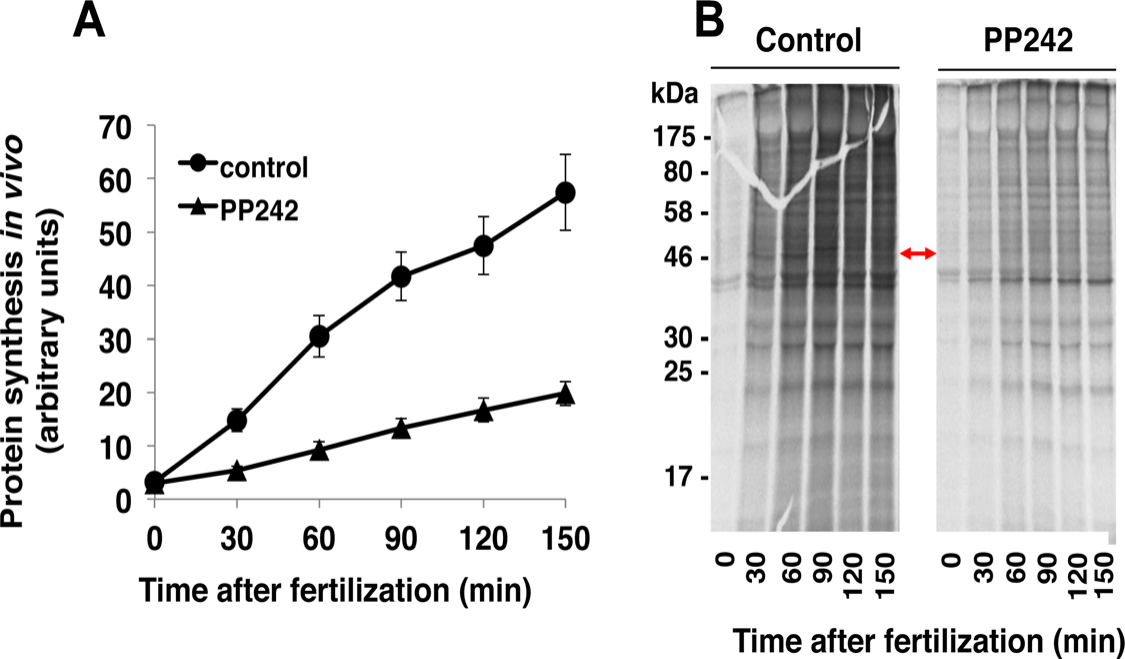
PP242 inhibits the increase in protein synthesis triggered by fertilization. **(A)** The rate of *in vivo* protein synthesis was monitored by the kinetics of [^35^S]methionine incorporation into proteins. *S*. *granularis* eggs (5% cells/vol solution in FSW) were metabolically labelled in the presence of [^35^S]methionine. After fertilization in the absence (circles) or presence (triangles) of 10 μM PP242, cytosoluble fractions were prepared from 20 μl pelleted embryos and radioactivity incorporation into TCA-precipitated proteins was determined at indicated times. Vertical bars represent Standard Error of the Mean values (SEM) obtained in 3 independent experiments **(B)** Pattern of proteins translated following fertilization of control (left panel) or PP242-treated (right) embryos. Proteins (30 μg) of the cytosoluble fractions were separated by SDS-PAGE (12%). Radioactive neo-synthetized proteins were visualized by autoradiography. Arrow shows subtle differences in the pattern of neo-synthetized proteins (see text).

Global protein synthesis activity was then compared in PP242-treated *versus* untreated embryos (Fig 3). In control embryos, as expected, fertilization induced an increase in the level of neo-synthesized proteins measured as indicated in Material and Methods. In contrast, in PP242-treated embryos, the neo-synthesized protein amount remained low, increasing only 4 fold from unfertilized eggs at 150 min after fertilization, as compared to the 10 fold increase for untreated embryos versus unfertilized eggs (Fig 3A). The profile of neo-synthesized proteins was analysed by one-dimensional SDS-PAGE analysis of the radioactive proteins. With the resolution limit of this method, no obvious significant difference in the level of specific proteins was observed following PP242 treatment (Fig 3B). However, it was noted that the behaviour of a 46/48-kDa neo-synthetized protein assumed to be cyclin (accumulation, shift at 90 min and disappearance at 120 min post fertilization) seemed not detectable in PP242-treated embryos (Fig 3B, arrow).

**Fig 4:**
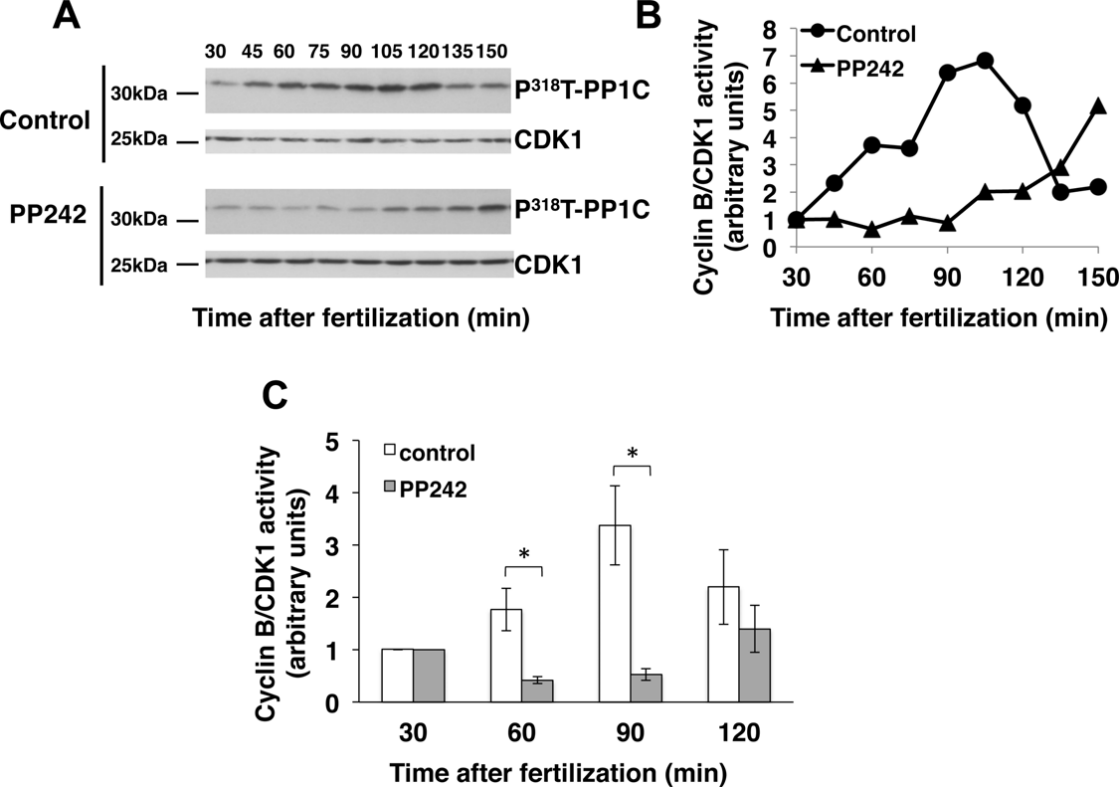
PP242 delays cyclin B/CDK1 activation triggered by fertilization. **(A)** *S*. *granularis* eggs were fertilized in the absence (control) or presence of 10μM PP242 (PP242). Aliquots of total extracts from 20 μl pelleted embryos taken at indicated times post-fertilization were resolved by 12% SDS-PAGE and subjected to Western blotting analysis using phospho-PP1Cα antibody (top panels). CDK1 immunolabelling with PSTAIR antibody was used as a loading control for Western blot (bottom panels). **(B)** Quantitation of the results obtained (in A) from control (circles) or PP242-treated triangles) embryos. Phospho^318^T-PP1Cα levels were normalized against CDK1 levels obtained at the same time and expressed as a ratio of the value obtained with unfertilized eggs. **(C)** Quantitation of Phospho^318^T-PP1Cα levels obtained from untreated (white boxes) and PP242-treated (grey boxes) eggs at the indicated time following fertilization. Phospho^318^T-PP1Cα level was normalized against CDK1 level and expressed as a percentage of the value obtained with unfertilized eggs. Vertical bars represent Standard Error of the Mean values (SEM) obtained in 6 independent experiments. (*) Indicates a significant difference in Phospho^318^T-PP1Cα level in the PP242-treated embryos in comparison to the untreated embryos (Wilcoxon signed-rank test, p<0.05).

To ascertain the cell cycle stage for the PP242 effect, the *in vivo* kinetics of cyclin B/CDK1 complex activation was monitored in treated embryos following fertilization. We took advantage of the specific phosphorylation of the alpha catalytic subunit of the serine/threonine-protein phosphatase 1 (PP1Cα) at threonine 320 (in the human sequence) by cyclin B/CDK1 [40] and the availability of antibodies that distinguish phosphorylated from non-phosphorylated PP1Cα at threonine 320 [38]. After validation of cross reactivity of the antibody on threonine phosphorylation site (^318^T in the sea urchin sequences) and validation of this assay in sea urchin (S1 Fig), cyclin B/CDK1 complex activation was monitored by Western blot analysis of the phospho^318^T-PP1Cα level in total extracts from embryos taken at different times after fertilization in the presence or not of PP242. Results from a typical experiment are reported (Fig 4A and 4B).

A peak of phospho^318^T-PP1Cα reflecting cyclin B/CDK1 activation appeared at 90 min post fertilization in accordance with the time of the first mitosis in untreated embryos. When embryos were treated with PP242, a significant and reproducible delay in cyclin B/CDK1 activation was observed (Fig 4C). Among 6 independent experiments the delay in cyclin B/CDK1 activation was of more than 90 min, correlating with the delay observed in cell division occurrence.

**Fig 5:**
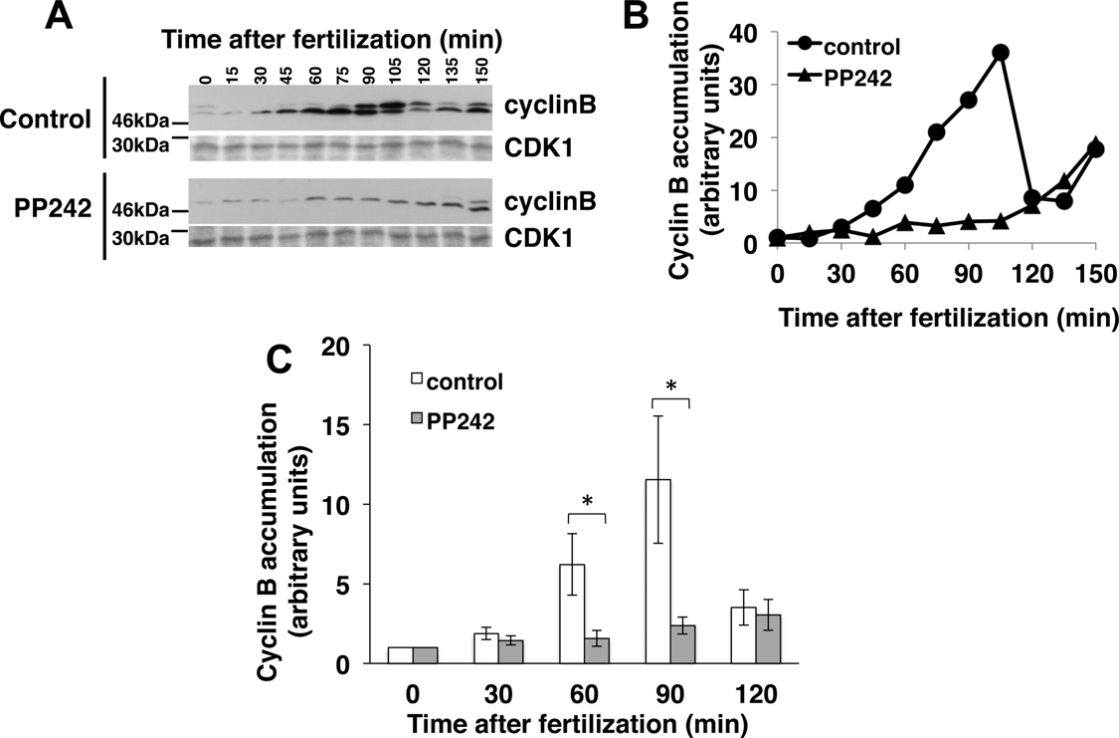
PP242 delays cyclin B protein accumulation triggered by fertilization. **(A)** Total amount of cyclin B in extracts from control or PP242-treated embryos of *S*. *granularis*, obtained at indicated times post-fertilization was monitored by Western blotting using anti-cyclin B (top panels). CDK1 immunolabelling with PSTAIR antibody was used as a loading control for Western blot (bottom panels). **(B)** Quantification of the results obtained from control (circles) or PP242-treated (triangles) embryos. Cyclin B level, the sum of the two immunorevealed bands, was normalized against CDK1 level and expressed as a ratio of the value obtained with unfertilized eggs. **(C)** Quantitation of cyclin B level obtained from untreated (white boxes) and PP242-treated (grey boxes) embryos at the indicated time following fertilization. Cyclin B level was normalized against CDK1 level and expressed as a percentage of the value obtained with unfertilized eggs. Vertical bars represent Standard Error of the Mean values (SEM) obtained in 6 independent experiments. (*) indicates a significant difference in cyclin B level in the PP242-treated embryos in comparison to the untreated embryos (Wilcoxon signed-rank test, p<0.05).

Considering the requirement for a critical amount of cyclin B to induce the formation of cyclin B/CDK1 complex [41, 42], we next addressed the dynamics of cyclin B production in the presence of PP242 (Fig 5). Cyclin B level was quantified in embryos by Western blotting at different times post-fertilization (Fig 5A and 5B). In PP242-treated embryos, cyclin B level did not increase before 135 min post-fertilization, delayed by more than 60 min compared to control embryos. This delay was reproducibly observed in 6 independent experiments (Fig 5C).

**Fig 6:**
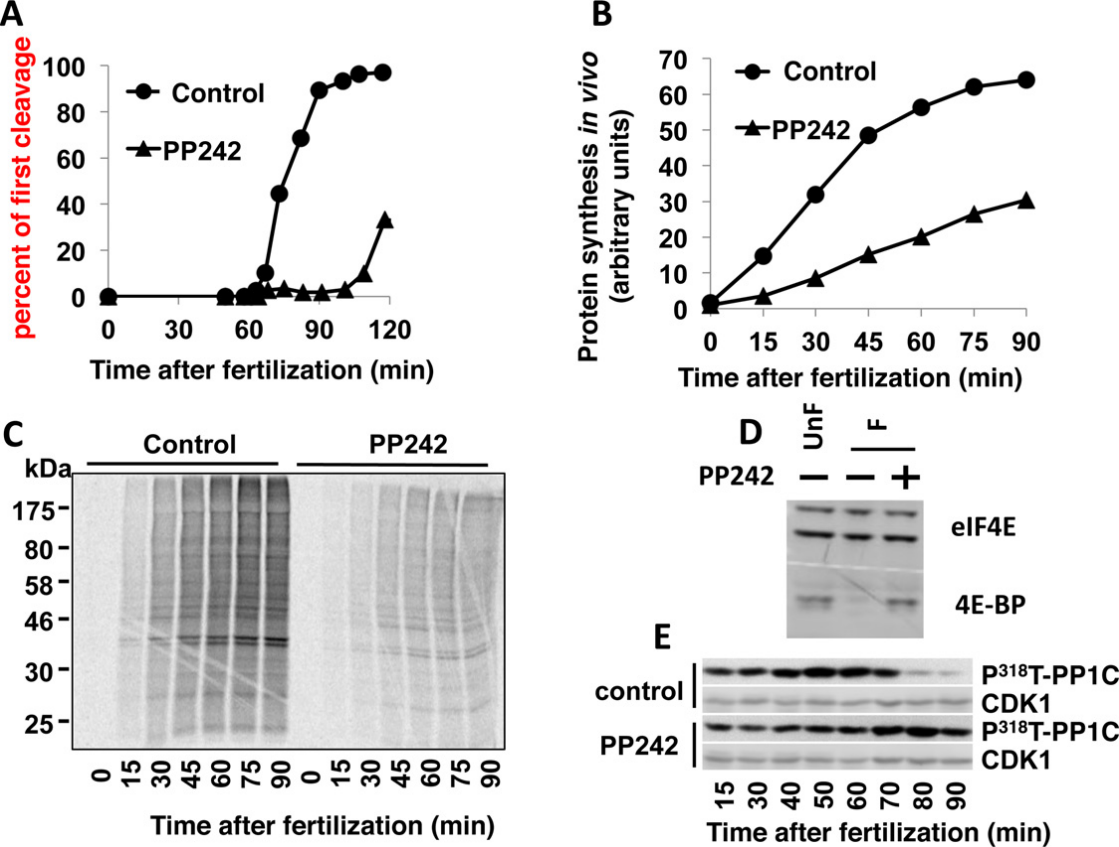
PP242 affects early embryonic development in *P*. *lividus*. **(A)** PP242 delays the first mitotic division triggered by fertilization. *P*. *lividus* eggs were incubated 10 min before sperm addition with DMSO (circles) or 10 μM (triangles) PP242. **(B)** PP242 affects the increase of protein synthesis. Embryos were metabolically labelled in the presence of [^35^S]methionine and the rate of *in vivo* protein synthesis was monitored in eggs fertilized in absence (circles) or presence of 10 μM PP242 (triangles). **(C)** Pattern of proteins translated following fertilization of control (left panel) or PP242-treated (right) embryos. **(D)** PP242 inhibits 4E-BP degradation triggered by fertilization. Total amount of 4E-BP in unfertilized eggs (UnF) or 60 min post-fertilized (F) embryos treated or not with PP242 was analysed by Western blotting using sea urchin 4E-BP antibodies (bottom panel). eIF4E immunolabelling with eIF4E antibody was used as a loading control (top panel). **(E)** PP242 affects CDK1/cyclin B activation triggered by fertilization. CDK1/cyclin B activation was monitored by Western blot analysis of the phospho^318^T-PP1Cα level. CDK1 immunolabelling with PSTAIR antibody was used as a loading control for Western blot. These figures are representative of 3 complete sets of experiments done with different females.

We carried out a similar set of experiments on embryos of *P*.*lividus*, a sea urchin species separated from *S*. *granularis* by 20 million years of evolutionary time. PP242 used at 10 μM delayed the first mitotic division (Fig 6A). Accordingly, the drug was found to affect global protein synthesis (Fig 6B and 6C), efficiently inhibit 4E-BP degradation (Fig 6D) and delay cyclin B/CDK1 complex activation (Fig 6E). Unfortunately, none of the available cyclin B-directed antibodies were found to cross-react with this protein in *P*. *lividus*, making the analysis of cyclin B protein expression after fertilization not possible (data not shown).

Altogether, these data demonstrated that, in two evolutionary distant sea urchins, a PP242-sensitive pathway is conserved and controls cyclin B accumulation. PP242 affects the degradation pathway of 4E-BP and consequently induces the stabilisation of the translational inhibitor, which maintains a low activity of protein synthesis, preventing cyclin B accumulation and cyclin B/CDK1 activation triggered by fertilization.

### PP242 inhibits *cyclin B* mRNA recruitment into polysomes following fertilization

**Fig 7:**
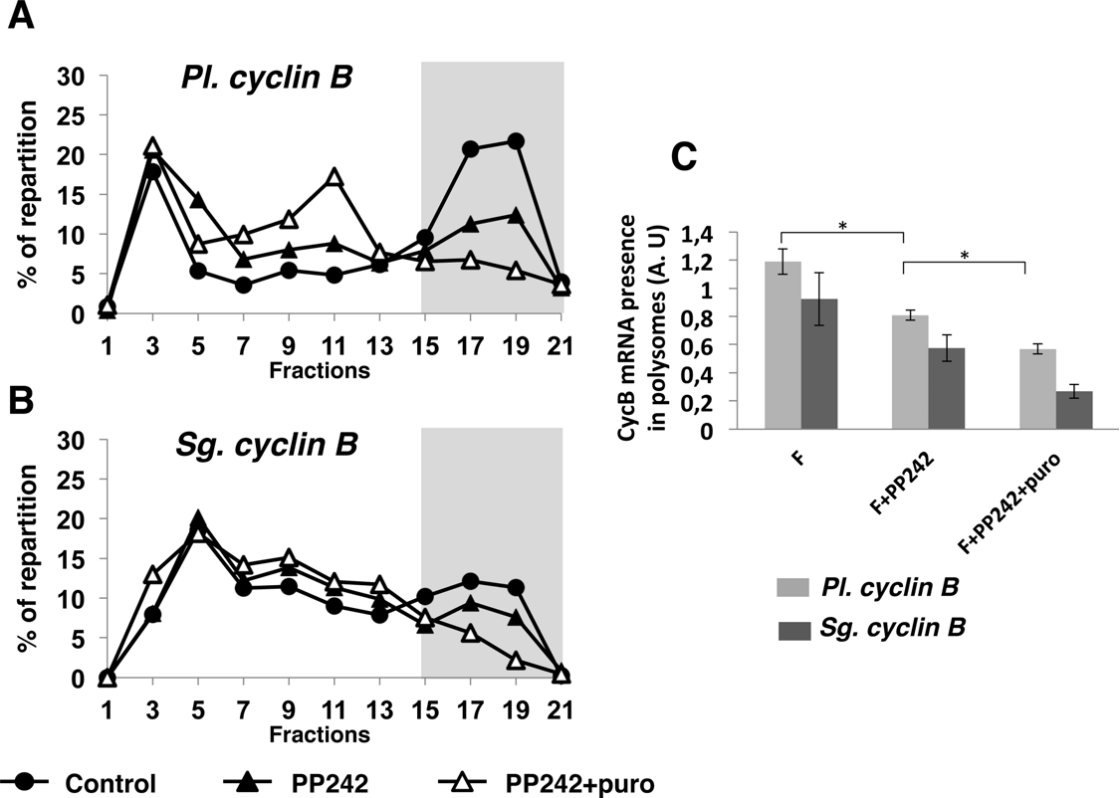
PP242 affects mitotic *cyclin B* mRNAs recruitment into active polysomes following fertilization in sea urchins. mRNAs were detected by RT-PCR amplification in each fraction of polysome gradients from control (filled circles), PP242-treated (filled triangles) or PP242-puromycin-treated (empty triangles) embryos. *P*. *lividus* and *S*. *granularis* embryos were respectively taken at 60 min and 90 min post-fertilization. Amplicons were run on agarose gels, quantified using ImageJ software and repartition was shown along the different fractions of the gradient as a percentage of total mRNA. Polysomal fractions are visualized by a grey box. **(A)** PP242 partially inhibits the recruitment of *Pl*. *cyclin B* mRNA into active polysomes (representative of six independent experiments: n = 6) **(B)** PP242 partially inhibits the recruitment of *Sg*. *cyclin B* mRNA into active polysomes (n = 3). **(C)** The area under the curve from fractions 15 to 21 was quantified. Significance for data obtained from six independent experiments using *P*. *lividus* eggs was assessed using Fisher’s F-test and Student’s t- test. * P<0.01.

Protein level is the result from its translation *versus* its degradation rates. Polysome analysis is widely used to evaluate changes in the translation status of specific mRNAs. [43, 44]. By optimizing a protocol to purify polysomes on sucrose gradient from sea urchin eggs, we showed that *cyclin B* mRNA became actively recruited into heavy fractions of a sucrose gradient following fertilization (S2 Fig, top panels). Puromycin, an inhibitor of the elongation step of translation, only impacts active polysomes and is used as a polysome disrupter to distinguish between translated mRNAs associated with polysomes and untranslated mRNAs co-migrating with polysomes because of their association to large ribonucleoprotein complexes [45]. Puromycin treatment of control embryos induced a complete shift of *cyclin B* mRNA from polysome fractions to monosome fractions, indicating that *cyclin B* mRNA was indeed recruited into active polysomes following fertilization (S2 Fig, bottom panels). We next compared *cyclin B* mRNA recruitment into polysomes in PP242-treated and untreated control embryos. We first chose to carry out those analyses using *P*. *lividus* embryos, which present more synchrony in the cell division kinetics. Polysome preparations from *P*. *lividus* embryos were performed at 60 min post-fertilization, corresponding to M-phase in control embryos as judged by the occurrence of the cyclin B/CDK1 activity peak (Fig 6E). As expected, *cyclin B* mRNA was actively recruited into the active polysomal fractions in control embryos (Fig 7A, black circles). In PP242-treated embryos, *cyclin B* mRNA associated to the heavy fractions significantly decreased (Fig 7A, black triangles), indicating that PP242 inhibited *cyclin B* mRNA polysomal recruitment. Furthermore, our data showed that the remaining amount of c*yclin B* mRNA in the PP242-treated embryos corresponded to active polysomes since it was shifted to lower fractions of the gradient after puromycin treatment (Fig 7A, empty triangles), suggesting a PP242-insensitive pathway. These data were reproducible in 6 different experiments (Fig 7C), and suggest two independent mechanisms for *cyclin* B mRNA recruitment. We also performed three experiments using *S*. *granularis* early embryos (Fig 7B). Polysomes of PP242-treated embryos were prepared at 90 min post-fertilization, at the time of M-phase in the control (Fig 2). As observed in *P*. *lividus*, *cyclin B* mRNA recruitment into active polysomes was partially inhibited by PP242 (Fig 7B and 7C).

Therefore we propose that *cyclin B* mRNA recruitment into active polysomes triggered by fertilization in both sea urchins is under the control of at least two different mechanisms, a PP242-sensitive pathway and a PP242-insensitive pathway.

## Discussion

In this study, we demonstrate that PP242 severely affected M-phase entry triggered by fertilization of sea urchin eggs. The initial target of PP242-induced mTOR inhibition would be the 4E-BP translational inhibitor, which remained undegraded in the presence of the drug. PP242 binds to the ATP-site of mTOR to inhibit both TORC1 and TORC2 [34]. 4E-BPs are well-characterized downstream targets of TORC1 (review in [27]). The present results confirm and extend our previous works [31, 46] showing that 4E-BP is degraded by mTOR-sensitive pathway triggered by sea urchin egg fertilization. A few reports [47, 48] had been published related to the control of 4E-BPs stability in mammals *via* ubiquitination and degradation by the proteasome. In mammal cells, ^57^Lys was the potential ubiquitination site in 4E-BP1 and only hypophosphorylated 4E-BP1 could be degraded, suggesting that 4E-BP degradation proceeds only when 4E-BP is not accessible to mTOR kinase activity. Therefore this mechanism could not account for the observed effect of PP242 in sea urchin embryo. Moreover, neither ^57^Lys nor ^69^Lys and ^105^Lys present in human 4E-BP1 are conserved in sea urchins [24, 49]. Thus it is likely that mTOR-mediated regulation of 4E-BP degradation relies to another molecular mechanism that remains to elucidate. Since many reports indicate that the 4E-BPs are functionally inactivated in cancer cells by major oncogenic signalling pathways [20], elucidation of the molecular mechanism involved in the mTOR-mediated regulation of 4E-BP degradation remains a major challenge. Our current work providing evidence that 4E-BP degradation depends on mTOR signalling confirms the strength of the sea urchin embryo as a model for the study of such mechanism.

Importantly, our data show that the fertilization-induced accumulation of cyclin B protein is repressed under mTOR pathway inhibition in two sea urchin species, *P*. *lividus* and *S*. *granularis*. We demonstrate that *cyclin B* mRNA recruitment into active polysomes is impacted by PP242 treatment of the embryos. This result definitely demonstrates that sea urchin egg fertilization stimulated the cap-dependent initiation of *cyclin B* mRNA translation *via* the mTOR-induced 4E-BP degradation.

In addition, our data further support two attractive hypotheses. On the one hand, the partial inhibition of *cyclin B* mRNA recruitment by PP242 treatment suggests that an additional mechanism, independent of 4E-BP level and insensitive to mTOR pathway, is involved in the recruitment of the *cyclin B* mRNA. This alternative pathway for *cyclin B* translation remains to be uncovered in sea urchin. Some reports have put forward 3’UTR-binding proteins in the control of *cyclin B* mRNA translation in different species (review in [3]). As an example, the RNA-binding protein CPEB (Cytoplasmic Polyadenylation Element Binding protein) binds specific sequence in the 3’UTR region of the messenger, determines polyadenylation status, and consequently translational efficiency, of its target mRNAs. In *Xenopus* oocyte, Maskin, which is a CPEB-interacting protein, sequesters eIF4E and supresses translation [50]. Therefore, Maskin serves as a classical example of a protein that links a RNA-binding protein to 3’UTR and eIF4E associated with the cap to inhibit translation initiation. Alternative mechanisms have since been identified involving an ovary specific eIF4E and the eIF4E-binding protein 4E-T [51]. CPEB-mediated control of cyclin B1 translation is critical for *Xenopus* embryonic division [4]. In starfish oocytes, we showed that cyclin B translation during meiotic maturation is correlated with the phosphorylation and the dissociation of CPEB from eIF4E [52]. Sea urchin contains genes involved in cytoplasmic polyadenylation such as CPEB, CPSF and Symplekin (review in [24]) and egg fertilization is associated with an increase in the polyadenylation of mRNAs [53]. However, it was reported [54] that sea urchin development prior to hatching is not affected by cordycepin, an inhibitor of RNA adenylation, suggesting that CPEB-mediated polyadenylation is not required for the first mitotic divisions in sea urchin.

In *Drosophila* ovaries and syncytial embryos, the PAN GU (PNG) kinase complex regulates the developmental translation of cyclin B [55]. PNG acts as an antagonist of PUMILIO-dependent translational repression, which represses cyclin B by binding the *cis*-acting Nanos Response Element (NRE) in the 3’UTR and by recruiting nanos [56]. Sea urchin embryos contain three nanos homologs. However, *nanos* mRNAs are only expressed in small micromeres [57], suggesting that the regulation of cyclin B translation following fertilization in sea urchin is independent of nanos.

Finally, Vasa, a broadly conserved ATP-dependent RNA helicase, has been reported to regulate the *in vitro* translation of *cyclin B* mRNA in a dual-luciferase assay in four- to eight-cell stage lysates of sea urchin embryos [58]. Since Vasa protein and mRNA are present uniformly throughout the egg and early embryo [59], Vasa could play a potential role in *cyclin B* mRNA translation regulation following fertilization.

Since a significant pool of *cyclin B* mRNA remains recruited and appears actively translated in the presence of PP242, a second exciting hypothesis drawn by our data came from the finding that cyclin B protein remains at a low basal level in presence of the drug (Fig 5). The intracellular level of a protein results from an equilibrium between its translation and its degradation rate. Therefore it can be assumed that the degradation of cyclin B protein must be activated in PP242 treated embryos in order to maintain a low level of cyclin B despite the mTOR insensitive recruitment of *cyclin B* mRNA triggered by fertilization. The simplest hypothesis to fit these data would be that fertilization triggers (i) the increase in *cyclin B* mRNA translation *via* both a mTOR dependent- and a mTOR independent-recruitment of *cyclin B* mRNA and (ii) the inhibition of cyclin B protein degradation *via* mTOR-signalling pathway.

Altogether our work brings important clues on the control of cyclin B translation *via* mTOR signalling and underlines the fine-tuning orchestration of mitotic cyclin translation after fertilization in sea urchin.

## Supporting Information

S1 Fig PP1Cα phosphorylation assay to monitor in vivo CDK1 activation in sea urchin embryos. (TIF)

S2 Fig Cyclin B mRNA is recruited into active polysomes following fertilization in sea urchins. (TIF)
