# Supplementary figures and images for: Cyclin B Translation Depends on mTOR Activity after Fertilization in Sea Urchin Embryos
Source: PLoS One. 2016 Mar 10;11(3):e0150318. doi: 10.1371/journal.pone.0150318 (PMC4786324; doi:10.1371/journal.pone.0150318)

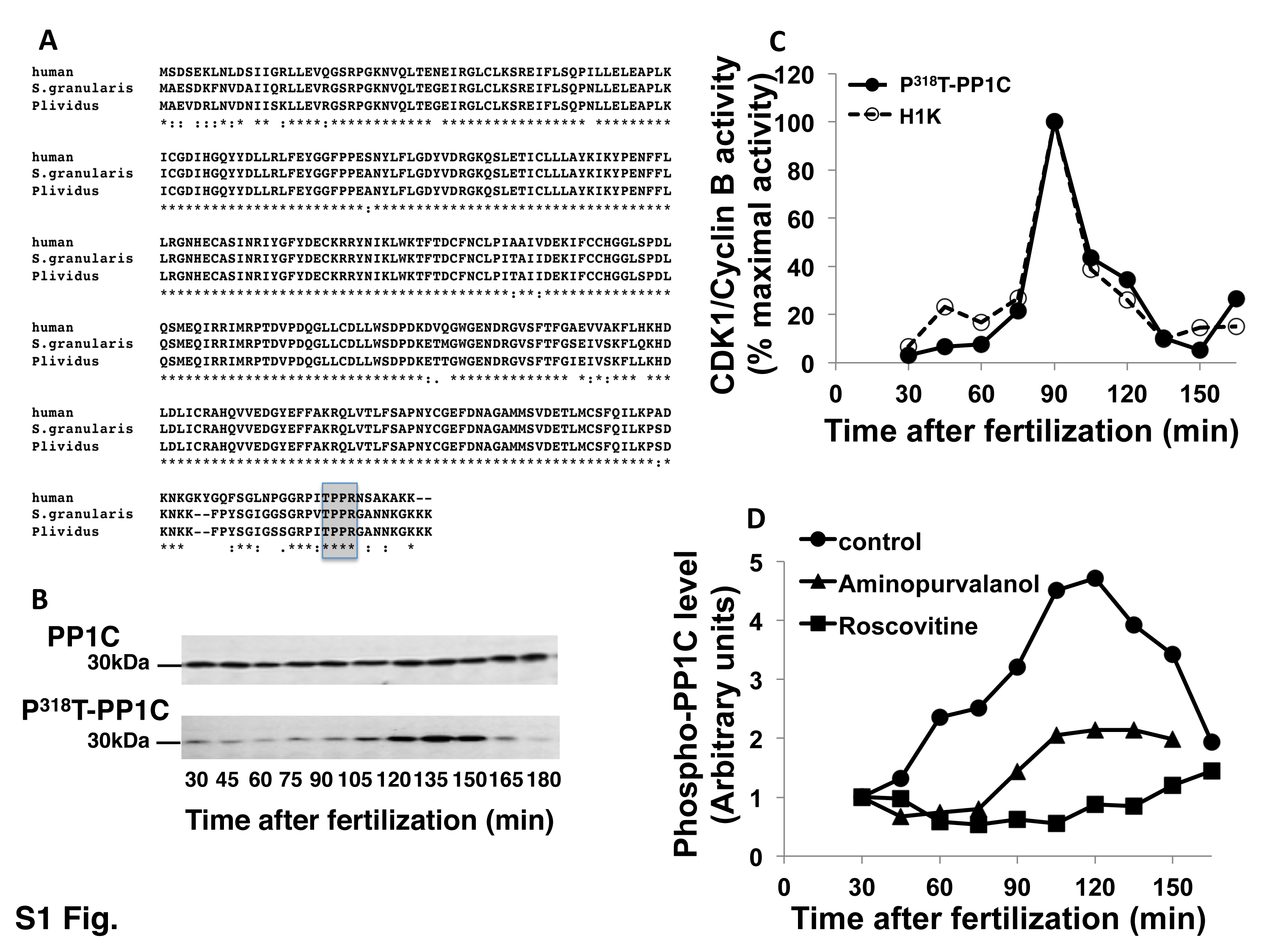

Supplement: S1 Fig — (TIF) [file pone.0150318.s001.tif]

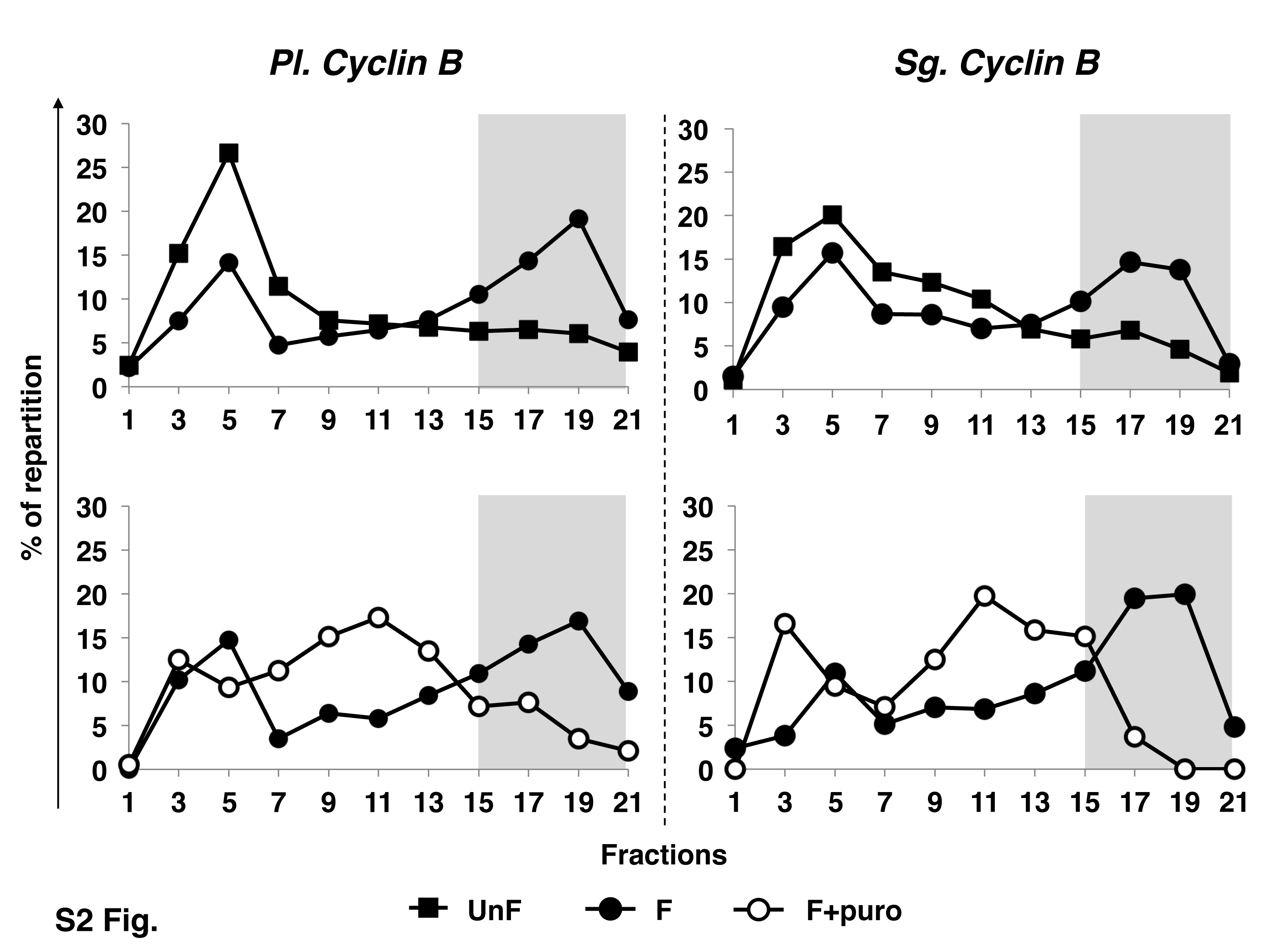

Supplement: S2 Fig — (TIF) [file pone.0150318.s002.tif]
